# (*E*)-Caryophyllene and α-Humulene: *Aedes aegypti* Oviposition Deterrents Elucidated by Gas Chromatography-Electrophysiological Assay of *Commiphora leptophloeos* Leaf Oil

**DOI:** 10.1371/journal.pone.0144586

**Published:** 2015-12-09

**Authors:** Rayane Cristine Santos da Silva, Paulo Milet-Pinheiro, Patrícia Cristina Bezerra da Silva, Alexandre Gomes da Silva, Marcia Vanusa da Silva, Daniela Maria do Amaral Ferraz Navarro, Nicácio Henrique da Silva

**Affiliations:** 1 Laboratory of Chemical Ecology, Department of Fundamental Chemistry, Federal University of Pernambuco (UFPE), Recife, Brazil; 2 Laboratory of Natural Products, Department of Biochemistry, Federal University of Pernambuco (UFPE), Recife, Brazil; 3 Institute of Experimental Ecology, University of Ulm, Ulm, Germany; Rosalind Franklin University, UNITED STATES

## Abstract

*Aedes aegypti* is responsible for the transmission of dengue, a disease that infects millions of people each year. Although essential oils are well recognized as sources of compounds with repellent and larvicidal activities against the dengue mosquito, much less is known about their oviposition deterrent effects. *Commiphora leptophloeos*, a tree native to South America, has important pharmacological properties, but the chemical profile and applicability of its essential oil in controlling the spread of the dengue mosquito have not been investigated. The aim of this study was to determine the composition of *C*. *leptophloeos* leaf oil and to evaluate its larvicidal and oviposition deterrent effects against *A*. *aegypti*. Fifty-five components of the essential oil were detected by gas chromatography (GC)—mass spectrometry, with α-phellandrene (26.3%), (*E*)-caryophyllene (18.0%) and β-phellandrene (12.9%) identified as the major constituents. Bioassays showed that the oil exhibited strong oviposition deterrent effects against *A*. *aegypti* at concentrations between 25 and 100 ppm, and possessed good larvicidal activity (LC_50_ = 99.4 ppm). Analysis of the oil by GC coupled with electroantennographic detection established that seven constituents could trigger antennal depolarization in *A*. *aegypti* gravid females. Two of these components, namely (*E*)-caryophyllene and α-humulene, were present in substantial proportions in the oil, and oviposition deterrence assays confirmed that both were significantly active at concentrations equivalent to those present in the oil. It is concluded that these sesquiterpenes are responsible, at least in part, for the deterrent effect of the oil. The oviposition deterrent activity of the leaf oil of *C*. *leptophloeos* is one of the most potent reported so far, suggesting that it could represent an interesting alternative to synthetic insecticides. The results of this study highlight the importance of integrating chemical and electrophysiological methods for screening natural compounds for their potential in combating vectors of insect-borne diseases.

## Introduction

*Aedes aegypti* (Linnaeus) is a known vector of various viruses including those responsible for yellow fever, dengue fever and chikungunya [1]. Among the neglected tropical diseases, dengue fever has shown the highest growth in prevalence worldwide with an estimated 30-fold increase over the last 50 years. According to the most recent estimates of the World Health Organization some 50 to 100 million people are infected with dengue each year [2], and this is particularly alarming because no effective vaccine has been developed so far [3]. Currently, therefore, control of the mosquito vector remains the principal method available through which to limit the dissemination of the dengue virus.

*Aedes aegypti* is a diurnal mosquito that is well adapted to urban areas, particularly in tropical regions where sanitation is poor [4]. Moreover, since the mosquito is able to breed wherever stagnant water accumulates (i.e. in discarded containers, plastic vessels, household water reservoirs, etc.), its control represents a challenging problem for public health authorities [5]. Historically, the main strategies for eliminating adults, larvae, pupae and eggs of *A*. *aegypti* involved the use of organochlorine (DDT, benzene hexachloride, dieldrin), organophosphorus, carbamate or pyrethroid insecticides [6]. However, the development of resistance by mosquitoes [7–10] necessitated more frequent application of these insecticides and continuous increase in dose rate. Thus, effective control of the mosquito became dependant on the limited number of synthetic insecticides available [11] and constrained by the deleterious effects of such compounds on the ecosystem and on human health [12].

Taken together, these aspects have alerted the scientific community to the need to develop new control strategies that would impede the rapid development of resistance by target vectors and mitigate the impact on the environment [13]. In this context, it is well known that plants have evolved a wide range of defensive compounds in order to protect themselves against herbivory [14], and that many of these natural products have low environmental persistence and limited toxicity to mammals. In this respect, strategies employing plant secondary metabolites represent promising alternatives in combating disease-transmitting insects [15].

Research interest in the application of plant essential oils in the fight against disease-transmitting mosquitoes stretches back some 25 years, with one of the first reports describing the repellent effect of *Tanacetum vulgare* (Asteraceae) oil on *A*. *aegypti* [16]. Since that time, numerous investigations have confirmed that essential oils have a broad applicability in controlling mosquitoes (including *A*. *aegypti*) by virtue of their repellent [17–19], attractant [20], deterrent [21,22] and larvicidal [23–26] properties.

While the capabilities of naturally occurring plant oils to combat disease-transmitting vectors have been well studied, data concerning the biological activities of individual components of an oil are generally limited to the major constituents [25,27,28]. However, compounds that are present in only very small amounts are frequently reported to play pivotal roles in insect behavior [29,30], although such components are frequently neglected. Gas chromatographic (GC) analysis coupled with electroantennographic detection (EAD) enables the identification of components of a complex matrix that have the ability to trigger receptor action potentials in the olfactory neurons of insect antennae [31,32] and, thereby, facilitates the selection of constituents with the potential to modify insect behavior.

Various reports are available [33–35] concerning the essential oils of members of the Burseraceae, a diverse plant family comprising some 500 species distributed among 20 genera [36]. The defensive roles played by a number of these essential oils have been investigated [35, 37–43], and repellent, insecticidal, anti-feedant, anti-infestation and oviposition deterrent activities have been demonstrated against diverse insect groups. In relation to disease-transmitting mosquitoes, the essential oil of *Canarium zeylanicum* has been shown to exert significant larvicidal effects against *A*. *aegypti*, *A*. *albopictus* and *Culex quinquefasciatus* [43], while that of *Commiphora molmol* is active against larvae of *Culex pipiens* [39].

*Commiphora leptophloeos* (Mart.) J. B. Gillet, a spiny deciduous tree that is native to South America, possesses many ethnopharmacological properties [44–47] and exhibits strong antimicrobial activity against *Staphylococcus epidermidis* [48]. However, the chemical composition of the essential oil of this species has not been reported and its activity against disease-transmitting mosquitoes is unknown. In the present study, we evaluated the potential of the leaf oil of *C*. *leptophloeos* as an environmentally friendly alternative for the control of *A*. *aegypti*. For this purpose, we performed a phytochemical investigation of the essential oil and investigated its larvicidal and oviposition deterrence activities against the dengue mosquito. Furthermore, we applied the GC-EAD technique to detect constituents of the leaf oil that are perceived by females, and tested selected components of the oil in biological assays.

## Methods

### Plant Material and Extraction of Essential Oils

Leaves of *C*. *leptophloeos* were collected in March 2012 at the Catimbau National Park, a nature reserve situated in the municipalities of Buíque, Ibimirm and Tupanatinga in the State of Pernambuco, Brazil. The authors confirm that the named authority “Instituto Chico Mendes de Conservação da Biodiversidade” granted permission (SISBIO 16806) for our described field studies. Voucher specimens were deposited in the Herbarium of the Instituto Agronômico de Pernambuco (IPA) with reference number 84 037. Essential oil samples were prepared in the Laboratory of Natural Products at the Department of Biochemistry, Federal University of Pernambuco (UFPE), by hydrodistillation of fresh leaves (150 g) for 4 h in a Clevenger apparatus. Oil samples were dried over anhydrous sodium sulfate, transferred to sealed vials and stored at -20°C until required for chemical analysis and biological assay [21,49].

### Maintenance of *A*. *aegypti* Population

A colony of *A*. *aegypti* of the Rockefeller strain was maintained in the laboratory at 28 ± 1°C under 70 ± 5% relative humidity and a 14 h photoperiod. Larvae were reared in plastic dishes and fed on a diet of commercial cat food (Whiskas^®^).

### Chemical Characterization of Essential Oils

Oil samples were analyzed by gas chromatography-mass spectrometry (GC-MS) using an Agilent Technologies (Palo Alto, CA, USA) model 7890A GC equipped with an Agilent J &W non-polar HP-5ms^™^ column (30 m x 0.25 mm id.; 0.25 μm film thickness) and coupled to an Agilent model 5975C mass selective detector. The analytical conditions were: oven temperature held at 40°C for 2 min then increased to 230°C at 4°C/min and subsequently held at 230°C for 5 min; helium flow maintained constant at 100 kPa; MS source set at 230°C; quadrupole temperature set at 150°C; mass spectra recorded at 70 eV in EI mode and scanned in the range *m/z* 35–350 at a speed of 0.5 s/scan. For each essential oil sample, a 1 μL aliquot of a solution containing 3000 ppm of oil dissolved in hexane was injected in split mode (1:20). Subsequently, 1 μL of a mixture of commercially available *n*-alkane standards (C9-C34; Sigma-Aldrich, St. Louis, MO, USA) in hexane was injected in split mode (1:20). Finally, 1 μL of a mixture comprising oil solution (0.2 μL) and hydrocarbon standards (0.8 μL) was injected in splitless mode. A retention index for each component of the essential oil was calculated according to the Van den Dool and Kratz equation [50] and compared with values reported in the literature [51]. The identities of constituents were verified by comparison of their retention times and mass spectral characteristics with those of authentic standards available in the MassFinfer4, NIST 08 and Wiley 9th Edition Registry reference libraries integrated into the Agilent MSD Productivity Chemstation.

### Electrophysiological Analyses

Leaf oil constituents potentially involved in the attraction/repellence of conspecific females were detected by electrophysiological analysis performed on a Thermo Scientific (Milan, Italy) Trace^™^ GC Ultra equipped with a Valco Instruments (Houston, TX, USA) ValcoBond^™^ VB column (30 m x 0.25 mm i.d.; 0.25 μm film thickness). The column outlet was fitted with a SGE Analytical Science (Trajan Scientific Americas, Austin, TX, USA) splitter tee connected to two lengths of deactivated capillary (40 cm x 0.25 mm i.d.). One capillary led to the flame ionization detector of the GC, while the second passed outside the GC oven into a glass tube where the effluent was mixed with a clean and humidified airflow and directed over mosquito antennae, the output signal of which was monitored by a Syntech (Kirchzarten Germany) EAD. The GC carrier gas was helium maintained at a constant flow rate of 1 mL/min, and nitrogen make-up gas was added to the column effluent before the splitter tee. For analysis, the oven temperature was set at 60°C and a 1μL aliquot of a solution containing 500 ppm of oil dissolved in hexane was injected in splitless mode with the injector temperature set at 200°C. After 1 min, the splitter tee valve was opened and the oven temperature increased at a rate of 7°C/min to 200°C and subsequently held at 200°C for 5 min.

In order to prepare mosquito antennae, 10 to 20 day old *A*. *aegypti* females were selected after the third day of blood meal, and heads were excised from thoraces using a scalpel. For each antenna preparation, the base of the head and the tips of both antennae were mounted between two glass capillary electrodes filled with insect ringer solution (8.0 g/l NaCl, 0.4 g/l KCl, 0.4 g/l CaCl_2_), the electrical circuit was completed with silver wire, and the antennal signal was amplified and passed to the interface of the EAD. In total, we prepared 50 antennae, of which only five were stable enough to detect responses. Leaf oil constituents that stimulated clear responses with at least three different antennae preparations were considered active.

### Oviposition Assays

Homogeneous solutions containing 25, 50 or 100 ppm of *C*. *leptophloeos* oil were obtained by dissolving 5, 10 or 20 mg, respectively, of leaf oil in 1.5 mL of co-solvent (acetone) and completing to 200 mL with distilled water. Test solutions containing α-phellandrene (26 ppm), (*E*)-caryophyllene (18 ppm), α-humulene (5 ppm) or terpinen-4-ol (0.14 ppm) were prepared in a like manner but with ethanol as co-solvent. In these assays, the concentration of each compound reflected the amount found in a preparation containing 100 ppm of *C*. *leptophloeos* leaf oil. Control solutions, prepared using the appropriate co-solvent but omitting test material, were included in all assays. The last oviposition bioassay was conducted using a mixture of (*E*)-caryophyllene (18 ppm), α-humulene (5 ppm) and terpinen-4-ol (0.14 ppm) in the test cups and the essential oil in the control cup.

Ten gravid *A*. *aegypti* females (10–20 days old) were transferred to a cage (30 x 30 x 20 cm) containing two disposable cups located 40 cm apart in diagonally opposite corners. Each cup was fitted with filter paper (12.5 x 12 cm) on the internal surface to provide support for oviposition, and one cup contained 25 mL of the test solution while the other held a similar volume of control solution. The cage was maintained in the dark at room temperature (28 ± 1°C) and 70 ± 5% relative humidity for 16 h, following which the oviposition response was determined by counting the numbers of eggs laid on the filter papers. For each assay, eight replicates were performed concurrently using eight separate cages. In order to verify the absence of influence of cage or location, additional bioassays were carried out in which both cups per cage contained equivalent control solutions.

The normality of the data obtained was verified using the Wilcoxon and Excel/Analyse-it normality tests. The mean numbers of eggs laid on filter papers in cups containing test solutions at various concentrations were compared with those of the respective paired controls using the Student’s *t*-test (MINITAB^®^ Release 14) at a *p*-level of 0.05.

### Larvicidal Assays

Homogeneous stock solutions containing 100 ppm of *C*. *leptophloeos* oil were prepared by dissolving 5.00 mg of leaf oil in 0.7 mL of co-solvent (ethanol) and completing to 50 mL with distilled water. Ethanol was chosen as co-solvent in this assay since it is not toxic to *A*. *aegypti* larvae at the concentration employed [21,26]. Preliminary test solutions were prepared at different concentrations (10, 50 and 100 ppm) and solution range (80–120 ppm) by appropriate dilution of the stock solution. Negative controls were prepared using the same amount of co-solvent but omitting the test material, while an aqueous solution of Temephos (1 μg/mL) served as positive control [26]. An adapted version [21] of the method recommended by the World Health Organization [52] was employed in order to evaluate median lethal concentration (LC_50_) values for larvicidal activity. Early fourth instar larvae of *A*. *aegypti* were transferred from the colony cage to disposable cups (20 larvae per cup) containing the test solutions and maintained under conditions that were identical to those of the colony. Four replicate assays were performed for every test solution at each concentration, and negative and positive controls were included in each assay. Larval mortality, taken as either a lack of response to mechanic stimulus or larvae not rising to the surface, was determined after 48 h, and LC_50_ values were calculated by Probit analysis using StatPlus 2008 software [21,49].

## Results

### Yield and Chemical Characterization of the Essential Oil

Hydrodistillation of *C*. *leptophloeos* leaves yielded 0.08% of essential oil. GC-MS analysis of the oil revealed 55 constituents, of which the 46 that could be unambiguously identified accounted for 97.8% of the total oil (Table 1). The major organic components were sesquiterpene hydrocarbons (mean 46.4%) followed by monoterpene hydrocarbons (43.4%), while the minor constituents were oxygenated sesquiterpenes (6.2%) and oxygenated monoterpenes (1.5%). The principal compounds in the leaf oil were α-phellandrene (26.3%), (*E*)-caryophyllene (18%), β-phellandrene (12.9%), germacrene-D (6%) and α-humulene (5.5%).

**Table 1:** Mean relative amounts of volatile compounds identified in the leaf oil of *Commiphora leptophloeos*.

		Retention index		Relative amount (%)
Peak	Constituents^a^	Calc.^b^	Lit.^c^	Mean ± SD
**1**	α-Pinene	932	932	1.41
**2**	β-Pinene	975	974	0.13
**3**	β-Myrcene	991	988	0.38
**4**	α-Phellandrene	1003	1002	**26.26**
**5**	(*Z*)-3-Hexenyl acetate	1008	1004	0.16
**6**	α-Terpinene	1016	1014	0.28
**7**	o-Cymene	1024	1022	1.36
**8**	β-Phellandrene	1028	1025	**12.93**
**9**	Eucalyptol	1030	1026	0.59
**10**	(*E*)-Ocimene	1049	1044	0.23
**11**	γ-Terpinene	1058	1054	0.20
**12**	α-Terpinolene	1088	1086	0.18
**13**	Linalool	1100	1095	0.41
**14**	Terpinen-4-ol	1177	1174	0.14
**15**	α-Terpineol	1190	1186	0.24
**16**	ð-Elemene	1337	1335	0.14
**17**	α-Copaene	1378	1374	0.18
**18**	Unknown 1	1387	-	0.06
**19**	β-Elemene	1394	1389	1.60
**20**	(*E*)-Caryophyllene	1423	1417	**18.01**
**21**	β-Copaene	1432	1432	0.29
**22**	Aromadendrene	1442	1439	0.18
**23**	Unknown 2	1444	-	0.14
**24**	(*E*)-Muurola-3,5-diene	1455	1451	0.16
**25**	α-Humulene	1458	1452	**5.46**
**26**	(*E*)-9-Epi-caryophyllene	1465	1464	2.26
**27**	γ-Muurolene	1481	1478	1.72
**28**	Germacrene D	1486	1480	**5.99**
**29**	β-Selinene	1491	1489	1.14
**30**	(*Z*)- β - Guaiene	1493	1489	0.15
**31**	ð-Selinene	1496	1492	0.45
**32**	α-Selinene	1500	1498	2.74
**33**	β-Alaskene	1501	1498	2.74
**34**	α-Muurolene	1505	1500	0.89
**35**	Germacrene A	1510	1508	1.51
**36**	γ-Cadinene	1519	1513	0.43
**37**	ð-Cadinene	1528	1522	2.33
**38**	Unknown 3	1530	-	0.15
**39**	Cadina-1,4-diene	1537	1533	0.13
**40**	α-Cadinene	1542	1537	0.10
**41**	Germacrene B	1561	1559	0.07
**42**	Palustrol	1571	1567	0.19
**43**	Unknown 4	1587	-	0.65
**44**	Unknown 5	1595	-	0.44
**45**	Cubenan-11-ol	1597	1595	0.40
**46**	Rosifoliol	1605	1600	0.27
**47**	Unknown 6	1616	-	0.25
**48**	Junenol	1622	1618	0.13
**49**	Unknown 7	1626	-	0.19
**50**	Unknown 8	1628	-	0.19
**51**	1-Epi-Cubenol	1631	1627	0.13
**52**	Unknown 9	1635	-	0.10
**53**	Epi-α-Muurolol	1645	1640	0.86
**54**	α-Muurolol	1649	1644	0.25
**55**	α-Cadinol	1657	1652	1.74
	Monoterpene hydrocarbons			43.36
	Oxygenated monoterpenes			1.54
	Sesquiterpene hydrocarbons			46.41
	Oxygenated sesquiterpenes			6.23
	Unknown			2.17
	**Total**			**99.71**

^a^ Constituents listed in order of elution on a non-polar DB-5 column;

^b^ Retention indices calculated from the retention times relative to a series of *n*-alkanes (C9-C19) analyzed on a DB-5 column;

^c^ Values obtained from Adams [51].

### Oviposition Assays

#### Assessment of the essential oil of *C*. *leptophloeos*

**Fig 1:**
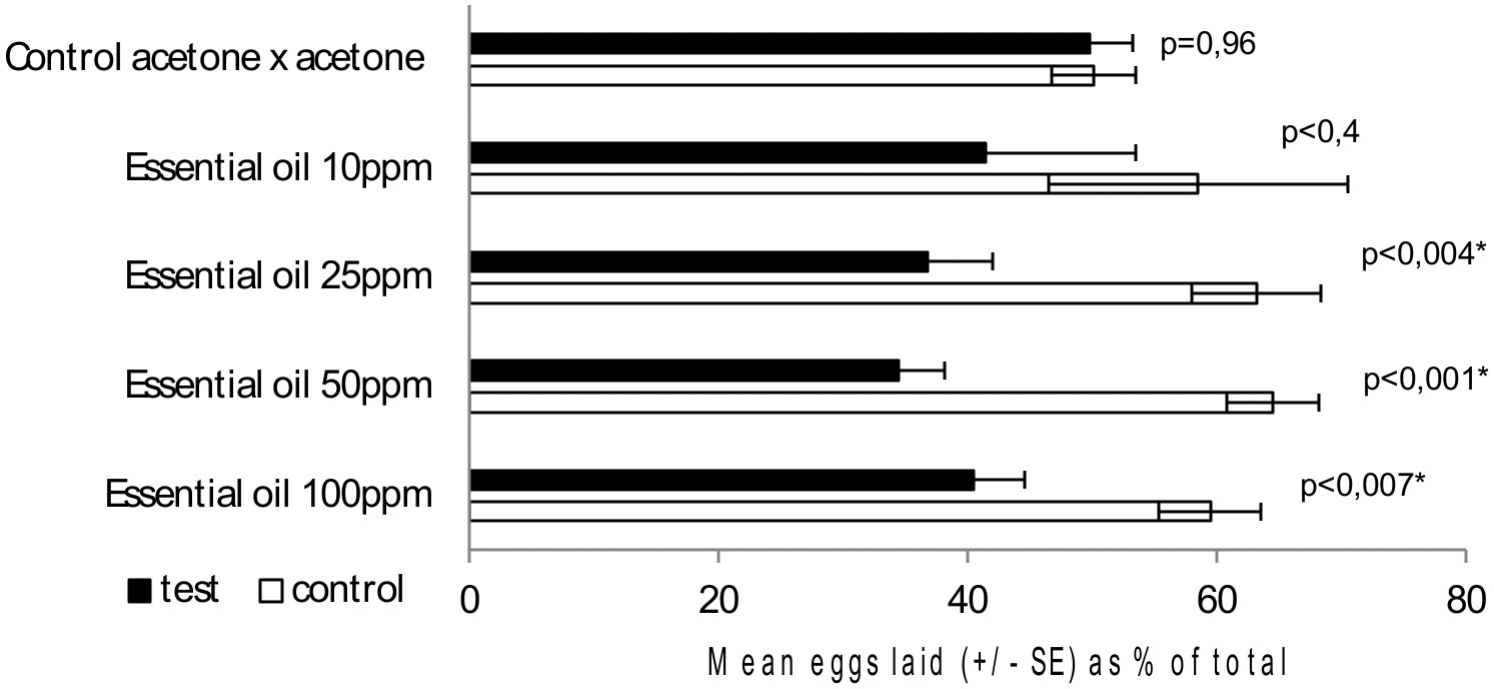
Relative mean numbers (±SD) of eggs laid by *Aedes aegypti* gravid females on filter papers treated with aqueous solutions of the essential oil of *Commiphora leptophloeos* at different concentrations or with appropriate control. Each assay was performed with ten mosquitoes and was replicated eight times. Mean values marked with an asterisk (*) are different at the level indicated (Student’s *t*-test).

Bioassays showed that, in comparison with respective controls, the presence of *C*. *leptophloeos* leaf oil in concentrations of 25, 50 or 100 ppm induced a reduction of more than 50% in the oviposition of *A*. *aegypti* gravid females (Fig 1). Additionally, bioassays involving control versus control verified that abiotic factors exerted no significant (*p* = 0.96) influence on oviposition.

#### Assessment of EAD-active components

**Fig 2:**
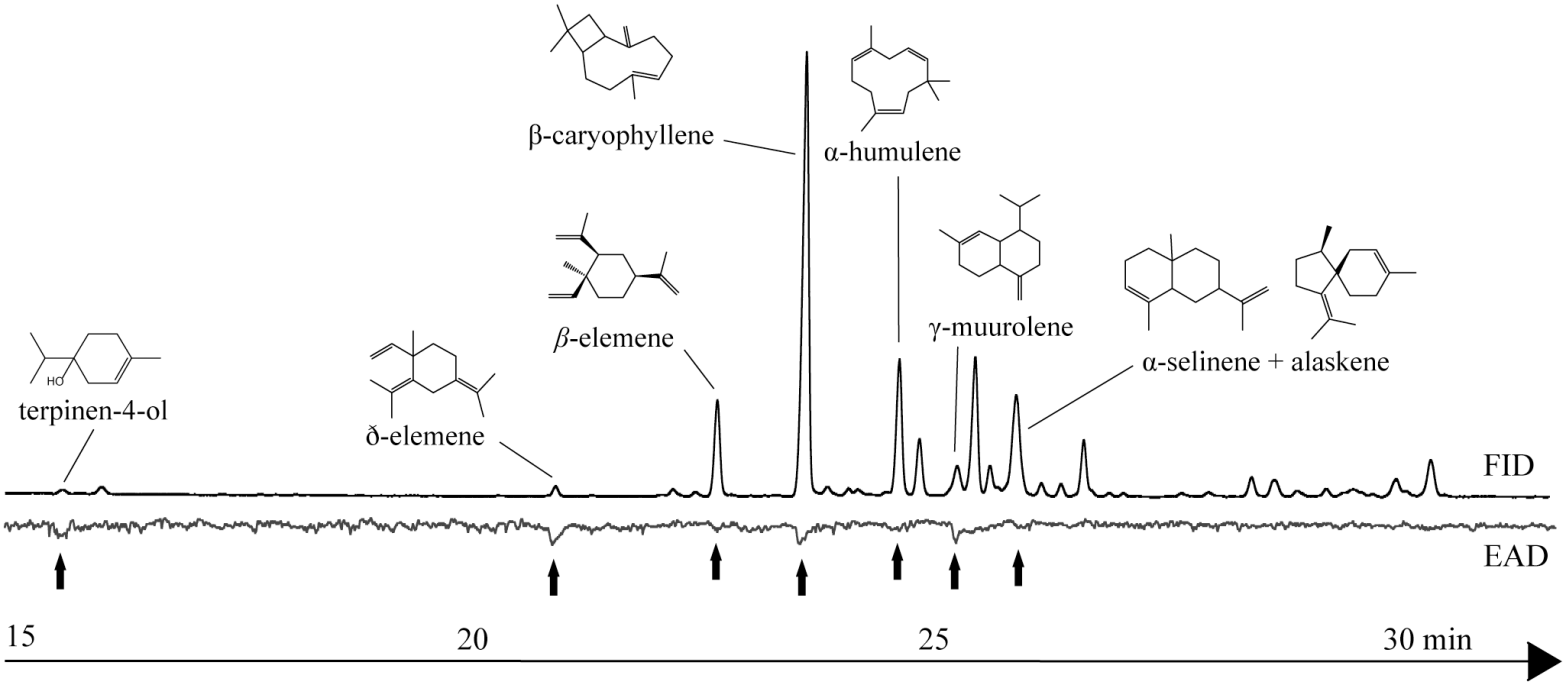
Antennal reactions of *Aedes aegypti* gravid females to components of the essential oil of *Commiphora leptophloeos* identified by gas chromatography coupled with electroantennographic detection.

**Fig 3:**
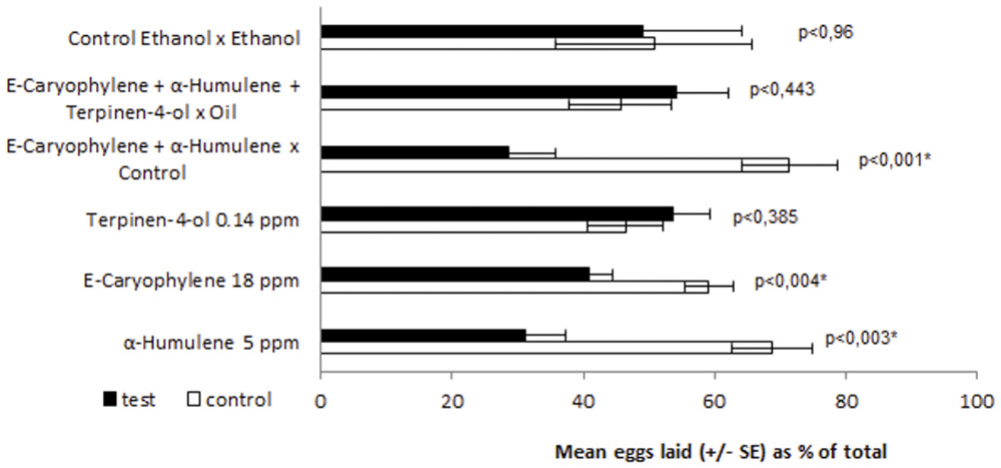
Oviposition responses of *Aedes aegypti* gravid females to aqueous solutions of the constituents of the essential oil of *Commiphora leptophloeos* identified by gas chromatography coupled with electroantennographic detection. The values represent the mean percentage (±SD) of the total eggs laid in response to the treatment. Each assay was performed with ten mosquitoes and was repeated eight times. Mean values marked with an asterisk (*) are different at the level indicated (Student’s *t*-test).

GC-EAD analysis of *C*. *leptophloeos* leaf oil demonstrated that one monoterpene (terpinen-4-ol), and the six sesquiterpenes (ð-elemene, β-elemene, (*E*)-caryophyllene, α-humulene, γ-muurolene, α-selinene/alaskene), triggered antennal depolarization in females of *A*. *aegypti* (Fig 2). Since selinene and alaskene eluted simultaneously, it was not possible to determine which of these two compounds elicited the antennal reaction and, therefore, both were considered active. The EAD-active sesquiterpenes (*E*)-caryophyllene and α-humulene featured among the principal components of the leaf oil and both compounds deterred oviposition by *A*. *aegypti* gravid females (Fig 3). Moreover, bioassay of a mixture containing the two sesquiterpenes (*E*)-caryophyllene and α-humulene) in proportions similar to those found naturally in the leaf oil revealed a subtle increase in deterrent effect in comparison with the bioassays in which compounds were tested individually. The monoterpene terpinen-4-ol, on the other hand, did not influence the oviposition behavior of females in the concentration tested (Fig 3), despite of having triggered antennal responses. A mixture composed of (*E*)-caryophyllene, α-humulene and terpinen-4-ol deterred oviposition as efficiently as the essential oil. The activity of the other EAD-active compounds was not tested because they were not available commercially.

The bioassay performed with the major constituent of *C*. *leptophloeos* leaf oil α-phellandrene, which was however not active electrophysiologically, resulted in no significant difference between the numbers of eggs deposited in the presence of the test samples and those laid in the control container (*p* < 0.2).

### Larvicidal Assays

The leaf oil of *C*. *leptophloeos* was active against early fourth instar larvae of *A*. *aegypti* and exhibited an LC_50_ value of 99.4 ± 2.7 μg/mL.

## Discussion

The leaf oil of *C*. *leptophloeos* was composed almost exclusively of C10 and C15 terpenoids of which monoterpene and sesquiterpene hydrocarbons comprised the major components, as is the case for most essential oils [53]. A number of studies of essential oils from species of *Commiphora* have shown that the chemical profiles of leaf oil within the genus are highly diverse [54–62]. In this context, the monoterpenes α-pinene, camphene, β-pinene, myrcene and limonene are widely distributed in different species, as are the sesquiterpenes β-elemene, α-copaene, α-humulene, β-selinene and germacrene B [54]. It is noteworthy that some components present in the oil from *Commiphora* species can also be found in members of the closely related genus *Bursera*. Thus, the sesquiterpenes α-phellandrene, β-phellandrene, (*E*)-caryophyllene and α-humulene identified in *C*. *leptophloeos* leaf oil have also been found in *Bursera copallifera*, *B*. *excelsa*, *B*. *mirandae*, *B*. *ruticola* and *B*. *fagaroides* var. *purpusii* [63]. These data are interesting because they point to other sources of active compounds similar to those found in the present study.

The antennae of *A*. *aegypti* are replete with chemoreceptors that enable the insect to detect air-borne stimuli and assist in locating suitable sites for oviposition [64]. However, volatile organic compounds have the capacity to promote or deter oviposition activity in *A*. *aegypti* females and, therefore, could have application in controlling the spread of viruses transmitted by this mosquito. Several studies have sought to determine the influence of essential oils on the behavior of mosquitoes. For example, Autran et al. [21] tested the essential oils from stems, leaves and inflorescences of *Piper marginatum* and showed that, in the presence of oils at concentrations of 50 and 100 ppm, *A*. *aegypti* females laid 40% fewer eggs in comparison with controls. Oviposition deterrence was also observed with the essential oil from inflorescences of *Alpinia purpurata* at a minimum concentration of 100 ppm, giving rise to a reduction of at least 50% in the number of eggs laid in test vessels in comparison with controls [49]. Our study revealed that the leaf oil of *C*. *leptophloeos* at concentrations of 25, 50 and 100 ppm exerted a strong effect on the oviposition of *A*. *aegypti* females, resulting in a reduction ranging from 59 to 63% in the number of eggs laid. On this basis, the leaf oil of *C*. *leptophloeos* represents one of the most effective deterrents against oviposition in *A*. *aegypti* reported so far for essential oils [18,22,65–67].

In *A*. *aegypti*, the oviposition behavior involves two main steps. First, the females evaluate potential oviposition sites using long-range cues, such as volatiles [68]. Once the oviposition site has been identified, short-range cues become increasingly important. Temperature and chemical signals received by contact chemoreceptors (e.g. gustatory receptors) are included as short-range cues. By using the GC-EAD technique, we were able to identified volatile compounds that might be involved in the long-range olfactory detection. The hyphenated GC-EAD technique allows the separation of constituents in a complex matrix and the subsequent detection of those that elicit depolarization in an insect antenna. Such depolarization represents the sum of the changes in action potentials in the olfactory neurons when stimulated by a given constituent, and strongly suggests (but does not establish) that the compound would mediate behavioral responses. However, the exact behavioral significance, if any, of an EAD-active constituent (e.g. attraction, deterrence etc) can only be established through appropriate bioassays [31,32,69].

Although oviposition deterrent activity of essential oils against *A*. *aegypti* has received considerable research attention, to the best of our knowledge, only one previous investigation has employed the GC-EAD technique to screen separated constituents for such effects. In this study, Campbell et al. [70] investigated the antennal responses of *A*. *aegypti* females to the essential oils of 11 plant species and reported a total of 42 EAD-active compounds, the most common of which were (*E*)-caryophyllene, linalool, 1,8-cineole, geraniol and geranial. However, these researchers did not verify the behavioral significance of the separated constituents.

In the present work, we integrated electrophysiological analyses with behavioral assays in order to establish the specific compounds responsible for the oviposition deterrent activity against *A*. *aegypti*. Seven components of *C*. *leptophloeos* leaf oil triggered antennal depolarization in gravid female mosquitoes and, of these, (*E*)-caryophyllene and α-humulene presented the highest abundance in the essential oil and were subjected to bioassay. When tested individually, both sesquiterpenes showed significant oviposition deterrent effects in that the mean numbers of eggs laid on filter paper soaked with (*E*)-caryophyllene and α-humulene, respectively, were 40.9 ± 3.7% (*p* < 0.004) and 31.2 ± 6.2% (*p* < 0.003) lower than the control. Furthermore, a binary mixture containing (*E*)–caryophyllene and α-humulene in proportions similar to those found in the essential oil produced a more accentuated reduction in oviposition than each compound individually, with 28.6 ± 7.3% of eggs posited in containers with test solution against 71.4 ± 7 3% laid in the control containers. These results, together with the fact that the mixture composed of EAD-active compounds was as deterrent as the essential oil, clearly show that (*E*)-caryophyllene and α-humulene act as oviposition deterrents (both individually and, to a greater extent, in admixture) and are responsible for the deterrent effect of the essential oil of *C*. *leptophloeos* against *A*. *aegypti* females.

(*E*)-Caryophyllene and its derivatives are widely distributed among plant oils, and reportedly possess acaricidal, insecticidal, repellent, attractive and antifungal properties [71–74]. The demonstration that the sesquiterpene also mediates behavior changes in *A*. *aegypti* leading to a reduction in oviposition suggests that the compound could be considered an excellent agent in controlling the spread of the dengue mosquito. Additionally, the present study has revealed for the first time that the presence of α-humulene gives rise to changes in mosquito behavior and exhibits high deterrent activity at low concentration (5 ppm), thus indicating a possible application in the control of *A*. *aegypti*. The practical use of (E)-caryophyllene and α-humulene could be to avoid mosquito oviposition in serendipitous breeding sites.

It is noteworthy that α-phellandrene, a major component of *C*. *leptophloeos* leaf oil, did not trigger antennal responses in the dengue mosquito during GC-EAD analysis, even though the sesquiterpene possesses strong larvicidal activity against *A*. *aegypti* and *A*. *albopictus* with LC_50_ values of 16.6 and 39.9 mg/mL, respectively [28]. It is likely, therefore, that *A*. *aegypti* females may not be able to detect compounds that are lethal to their offspring, as suggested previously by Autran et al. [21]. In order to confirm this possibility, we carried out oviposition assays with α-phellandrene and found that the sesquiterpene has no significant oviposition activity against *A*. *aegypti* females. Furthermore, we established that the essential oil of *C*. *leptophloeos* is active against early fourth instar larvae of the mosquito (LC_50_ of about 99 μg/mL), indicating that the larvicidal activity of the leaf oil may be attributed partially (but not uniquely) to the presence of α-phellandrene. These findings are significant with regard to combating the spread of *A*. *aegypti* and of other disease-transmitting insects. The use of compounds such as α-phellandrene that are lethal to larvae but not detectable by adults could be employed in conjunction with traps designed to attract ovipositing females but prevent the subsequent development of larvae.

Moreover, the results presented herein verify that components isolated from essential oils can be applied as natural alternatives in controlling the spread of *A*. *aegypti* by preventing the deposition of eggs near human habitations and by acting as larvicidal agents.
